# Should routine risk reduction procedures for the prevention and control of pandemics become a standard in all oncological outpatient clinics? The prospective COVID-19 cohort study: protect-CoV

**DOI:** 10.1007/s12032-022-01700-4

**Published:** 2022-04-10

**Authors:** Theres Fey, Nicole Erickson, Arndt Stahler, Maximilian Muenchhoff, Oliver T. Keppler, Katharina Ruehlmann, Gabriele Krauss-Pfeiffer, Hannah Steinberg, Alexander Graf, Stefan Krebs, Helmut Blum, Elham Khatamzas, Sarah Seynstahl, Jozefina Casuscelli, Daniel Markwardt, Roswitha Forstpointner, Timo Schinköthe, Michael von Bergwelt-Baildon, Volker Heinemann

**Affiliations:** 1 Comprehensive Cancer Center (CCC Munich LMU), Ludwig Maximilians University (LMU) Hospital, Munich, Germany; 2 Department of Hematology, Oncology and Tumor Immunology, Charité – Universitätsmedizin Berlin, Corporate Member of Freie Universität Berlin and Humboldt-Universität zu Berlin, Charitéplatz 1, 10117 Berlin, Germany; 3 Max von Pettenkofer Institute & Gene Center, Virology, National Reference Center for Retroviruses, Ludwig Maximilians University München, Pettenkoferstr. 9a, 80336 Munich, Germany; 4 German Center for Infection Research (DZIF), Partner Site, Munich, Germany; 5 Laboratory for Functional Genome Analysis, Gene-Center, Ludwig Maximilians University, Munich, Germany; 6 Center for Infectious Diseases, Heidelberg University Hospital, Heidelberg, Germany; 7 Department of Urology, Ludwig Maximilians University (LMU) Hospital, Munich, Germany; 8 Department of Gastroenterology, Ludwig Maximilians University (LMU) Hospital, Munich, Germany; 9 Department of Oncology and Hematology, Ludwig Maximilians University (LMU) Hospital, Munich, Germany; 10 CANKADO Service GmbH, Cologne, Germany

**Keywords:** Covid-19, SARS-CoV-2, Immunology, Cancer, Pandemic, Outpatient

## Abstract

Limited knowledge exists on the effectiveness of preventive preparedness plans for the care of outpatient cancer patients during epidemics or pandemics. To ensure adequate, timely and continuous clinical care for this highly vulnerable population, we propose the establishment of preventive standard safety protocols providing effective early phase identification of outbreaks at outpatient cancer facilities and communicating adapted standards of care. The prospective cohort study Protect-CoV conducted at the LMU Klinikum from mid-March to June 2020 investigated the effectiveness of a rapid, proactive and methodical response to protect patients and interrupt SARS-CoV-2 transmission chains during the first pandemic wave. The implemented measures reduced the risk of infection of individual cancer patients and ensured safe adjunctive infusion therapy in an outpatient setting during the early COVID-19 pandemic. In addition to the immediate implementation of standard hygiene procedures, our results underscore the importance of routine PCR testing for the identification of asymptomatic or pre-symptomatic COVID-19 cases and immediate tracing of positive cases and their contacts. While more prospective controlled studies are needed to confirm these results, our study illustrates the importance of including preventative testing and tracing measures in the standard risk reduction procedures at all out patient cancer centers.

## Introduction

The coronavirus SARS-CoV-2 was first documented in Wuhan, China in November 2019. Thereafter, it rapidly spread worldwide leading the Director-General of the World Health Organization (WHO) to declare its highest level of alarm announcing a public health emergency of international concern (PHEIC) on January 30th 2020 [1]. As the pandemic raged on and scientists raced to develop appropriate therapeutics and vaccines, it became important to address the impact this rapidly spreading viral infection was having on patients receiving medical care for high-risk conditions. Cancer patients who are receiving systemic cancer treatments are considered to have a particularly increased risk of breakthrough infections and severe disease. Even after vaccine rollouts, cancer patients remain among such high-risk populations—in part due to the immunosuppressive nature of many treatment regimes [2–4]. Furthermore, health-care workers have a higher risk of infection compared to the average population given their increased daily patient contact within the context of direct patient care, and can also serve as potential disease vectors if they become infected [5].

COVID-19 is characterized by rapid airborne transmission and can therefore potentially spread quickly throughout an outpatient cancer ward. However, data regarding the effects of pandemics on outpatient cancer settings, particularly haemato-oncological clinics are limited. The risk of exposure for patients and health-care workers in such settings during pandemics remains mostly unknown. Descriptions of specific risk management procedures aimed to proactively protect such vulnerable populations, as well as the health-care workers who treat these patients, are also rare. Even as the vaccine rollout contributes to slowing the spread of the pandemic, viral variants are still emerging and breakthrough infections have been documented among the more vulnerable populations [6].

Combined with the documented mental stress and uncertainty toward management of infected patients and health-care workers alike, it is therefore important that risk reduction guidelines go beyond those in place for the general healthy population [7]. Although different variations of risk reduction measures have been implemented worldwide, proactive non-symptomatic risk reduction measures which include non-symptomatic testing requirements for both patients and health-care workers is still not standard practice. Therefore, the aim of this cohort study was to evaluate the effectiveness of routine risk reduction procedures, including non-symptomatic testing in an oncological outpatient clinic.

## Research in context

The first case of COVID-19 detected in Germany was documented on January 27th 2020. This case series became known as the Bavarian cluster and represented the first documented human transmission of SARS-CoV-2 outside of Asia. Böhmer et al. later presented data linking this case to 16 other cases beginning in China and reaching as far as Spain. The secondary attack rate was calculated to be 5.1% and a short incubation period was confirmed [8]. Seven weeks later, on March 16th, Germany reported 6012 confirmed cases and 13 deaths. The state of Bavaria reported 7.4 cases per 100,000 residents. Eight countries, or regions thereof were classified as risk areas, social distancing measures were enforced, and schools and cultural institutions were closed. In addition gatherings of over 1000 people were not allowed. Masks were first required at the end of April and here the requirements were limited to inside shops and public transport. By this point however, most hospitals and health-care institutions were recommending masks in areas with certain patient populations [9].

By March 18th, 8198 COVID-19 cases and 12 deaths had been confirmed in Germany [10]. It was against this backdrop of events that on March 18th 2020, a nurse employed in the outpatient cancer treatment center presented with a sudden onset of fever. Management and investigation of the outbreak was immediately initiated. Four weeks later a prospective data collection began with the goal of implementing and evaluating strategies for risk reduction in such settings.

## Materials and methods

This prospective registry study was designed to observe the effectiveness of a preventative and regular test strategy combined with hygiene measures aimed to protect both a vulnerable patient population and health-care professionals working in a haemato-oncological outpatient cancer clinic at the Ludwig Maximilians University Hospital of Munich in Germany. On average about 40 patients receive intravenous cancer-specific therapies at the outpatient clinic per day and these are delivered in one of eight rooms measuring 28 square meters each. The multidisciplinary team employed in the outpatient clinic consists of 28 members in total (e. g. physicians, nurses, study nurses, dietitians, and administrative staff). This study was approved by the Institutional Ethics Committee at the Ludwig Maximillian University of Munich in Germany (Reference number 20-360) and informed consent was provided by all subjects.

### Phase I: description of outbreak and tracing

The primary case was immediately isolated and tested positive for SARS-CoV-2 by real-time reverse-transcription polymerase chain reaction assay test (RT-PCR) via a nasopharyngeal swab sample [11, 12]. For all patients and staff members with contact to the index case, subsequent testing by real-time quantitative PCR (RT-qPCR) and isolation was recommended. All health-care providers (HCPs) were immediately required to wear a medical mask. Thereafter, infection chain identification supported by SARS-CoV-2 sequence analyses was performed and recorded.

### Phase II: introduction of measures to protect staff and patients

Four weeks after the first case, a rigorous system of prospective data collection consisting of regular tests was administered to patients and HCPs and continued prospectively for 2 months. All HCPs with patient contact performed bi-weekly RNA-PCR assay tests. Patients underwent weekly PCR screening. Furthermore, serological data was obtained monthly to detect the presence of IgA and IgG antibodies. Additionally, measures were taken to ensure that all people on the ward were able to practice social distancing as per regulations. For example, the distance between the patient chairs was expanded. Lastly, an app-based digital diary designed by CANKADO to enable early identification was utilized among a subset of the patients who agreed to download and use the application. The app is designed to track symptoms commonly associated with Covid-19 and provide recommendations regarding what steps the patients should take according to the severity of symptoms recorded.

### Real time polymerase chain reaction (RT-PCR)

Nasopharyngeal swab specimen were tested for SARS-CoV-2 RNA in the accredited diagnostic laboratory at the Pettenkofer Institute, Munich. Different PCR assay systems were used as described previously [13].

### Virus genome sequencing and tracing

Whole virus genome sequencing Amplicon pools from ARTIC multiplex PCR spanning the SARS-CoV-2 genome were prepared for each sample, converted to barcoded sequencing libraries with the Nextera XT kit (Illumina, San Diego USA) and sequenced on a Illumina Hiseq1500 sequencer [14]. The sequenced amplicons were demultiplexed and consensus sequences were generated using the *iVar* pipeline [15]. Briefly, the short reads were mapped against the SARS-CoV-2 reference genome (NC_045512.2) with *bwa-mem *[16]. The reads were first filtered with *iVar trim* using default parameters which were used to generate pileup files with *samtools* [17]. The pileup files served as input for the consensus sequence generation within *iVar* where only variants that had a minimum read depth of 20 and a minimum frequency of 0.9 were considered. Phylogenetic analyses were achieved with the web- and analysis platform Auspice using the SARS-CoV-2 build (https://github.com/nextstrain/ncov) and the bioinformatic toolkit augur [18, 19]. The consensus sequences and meta data for the samples were uploaded to the GISAID repository.

**Fig. 1:**
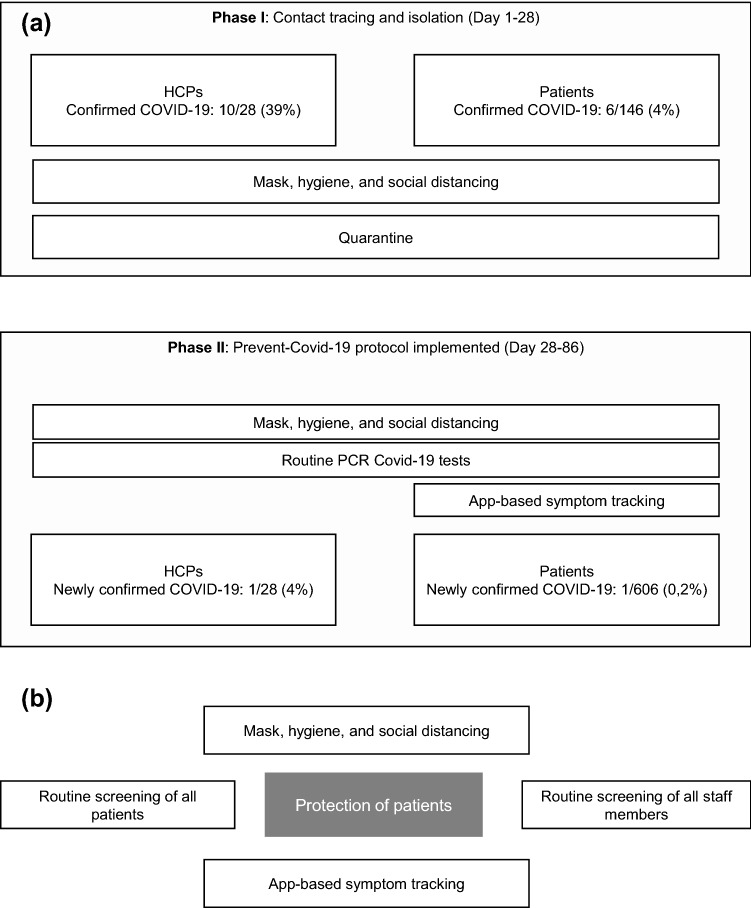
**a** Overview of the COVID-19 outbreak and contact tracing. **b** Prevent-Covid-19 protocol

Serological testing was performed at the accredited laboratories at the Pettenkofer Institute. In total, data were acquired and analyzed from 28 HCP and 606 patients (Fig. 1a, b).

### CANKADO’s digital Covid-19 App

The digitally based Covid-19 App developed by CANKADO was utilized in a subset of the population. CANKADO provides full patient privacy protection and data handling compliant with ICH GCP E6(R2). CANKADO is approved as an active Class I medical device within the European Union (registration number DE/CA59/11976/2017).

## Results

### Phase I: description of the outbreak and contact tracing

Following the detection of the index case, all patients who had contact with the index case for up to 48 h before symptom onset of the index case were alerted. In total, 146 patients were contacted and it was recommended that they self-isolate for 14 days. These patients were instructed to get a PCR-Test at the onset of any symptoms known to be associated with SARS-CoV-2 such as fever, cough, runny nose, headache, sore throat etc. All HCPs involved in patient care, or who had contact with the index case, were also offered voluntary tests. A subset of the subjects who tested positive for SARS-CoV-2 agreed to share data related to virus genome sequencing (*n* = 6 HCPs, *n* = 3 patients) and another subset (*n* = 8 HCPs) agreed to provide information regarding symptoms and disease progression. In total, 10 (10/28, 36%) staff members tested positive for SARS-CoV-2 within the first 2 weeks after contact with the index case. Four percent (6/146) of the patients who received treatment at our outpatient clinic also reported positive tests for SARS-CoV-2 during the initial 4-week period (Fig. 1a). Within two days of the initial diagnosis of the index patient, six HCPs had positive PCR-tests. Another four staff members tested positive within 1 week after the index case was diagnosed. Among the personnel who tested positive, two were physicians, six were nurses, one was a study nurse, one belonged to the cleaning staff, and one belonged to the administrative staff. Virus genome sequencing and phylogenetic analysis was performed on 5/9 HCPs and 3/6 patients who tested positive during this period.

**Fig. 2:**
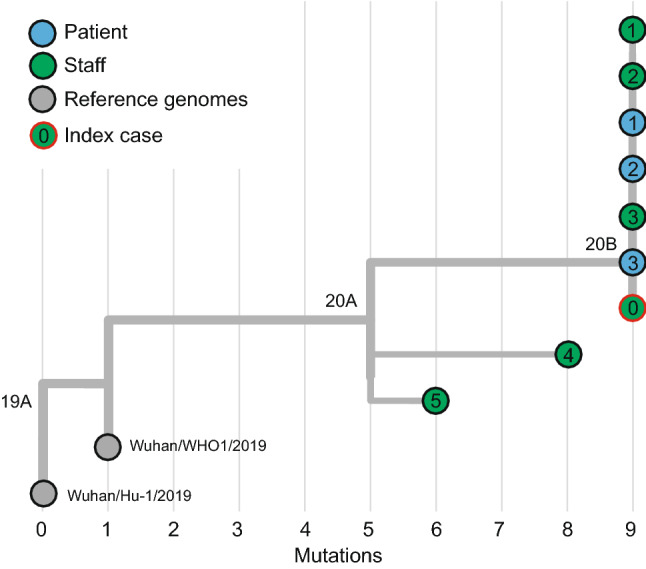
Virus genome sequencing and phylogenetic analysis of positive COVID-19 tests

Results of the phylogenetic analyses revealed that six of these eight cases were linked to the index case (staff 0) showing identical virus sequences (staff 1–3 and patients 1–3, Fig. 2). The two remaining cases (staff 4 and 5) were genetically distinct by several single nucleotide polymorphisms (SNPs) and thus determined to be unrelated to the index case.

### Phase II introduction of measures to protect staff and patients

In phase II, a total of 890 RNA-PCR assays were performed on a total of 606 patients in a 9 week period during stretching out over the months of April to June 2020.

Two patients (0.3%) tested positive during this phase, but only one was considered to be a new case. Among the 28 HCPs, a total of 167 RNA-PCR assays were performed of which four (1.6%) were positive. Only one of these positive results was a new case.

Serological data was collected from both staff members and patients in order to detect potential asymptomatic and undiagnosed infections by assessing longitudinal seroconversion.

Samples were collected from 525 patients and 77 HCPs. All HCP without a previous diagnosis of SARS-CoV-2 infection tested negative for both IgA and IgG antibodies with the exception of one. This HCP never tested positive for SARS-CoV-2 by PCR, yet showed IgA and IgG antibodies against SARS-CoV-2. Among the HCPs who tested positive for SARS-CoV-2 by PCR, all showed both IgA and IgG antibodies, again with the exception of one whose serum did not show IgG antibodies during the whole period of testing (80 days).

### Disease presentation and treatment

Three of the six infected patients provided more specific information regarding the course of their disease. Two of these three patients required hospitalization due to their SARS-CoV-2 infection. One patient spent a total of 51 days in the intensive care unit and required mechanical ventilation. None of these patients died.

**Fig. 3:**
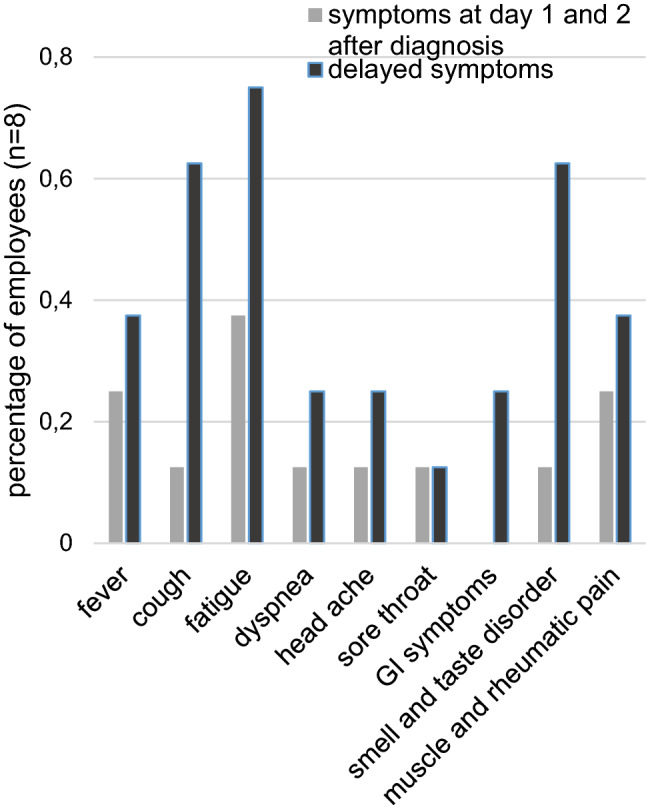
Symptoms experienced among affected staff

Symptom tracking among staff members revealed that seven out of the eight staff members who were tested positive for SARS-CoV-2 presented initially with relatively mild symptoms. The remaining staff member who tested positive was asymptomatic. In fact, five of the eight staff members experienced such mild symptoms they did not realize they were infected and reported to work initially. As the disease progressed, symptoms increased including fever, coughing, fatigue, dyspnea, headache, sore throat, dysgeusia, muscle and joint pain and gastrointestinal symptoms. Fatigue, coughing, and dysgeusia occurred most frequently (Fig. 3). One staff member experienced no symptoms at all.

During Phase II, 39 of the 525 patients (7%) also chose to download and use the symptom tracker per app. A detailed analysis of the utility of the app in this setting providing further data and details is in progress.

### Socio-economic burden

**Fig. 4:**
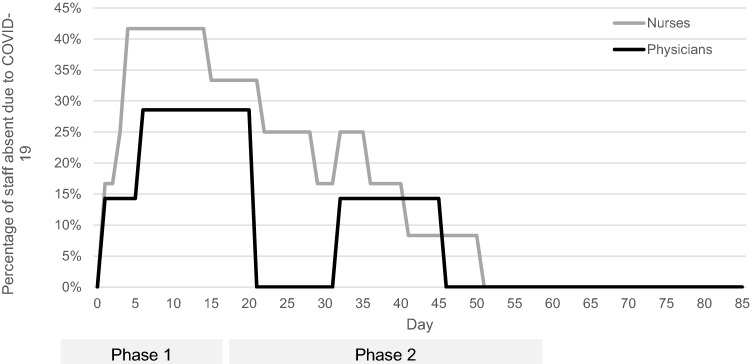
Total average duration of leave required among staff affected by COVID-19

Sick day leave due to Covid-19 was also tracked carefully among all 11 staff members who had close patient contact. In total, the outbreak caused 219 days of sick leave spread throughout the affected members of staff. During this time, up to 42% of the nursing staff and 29% of the physicians were simultaneously absent for at least a 10 day period. The total average duration of leave was 20 working days (range 13–32 days) (Fig. 4). The personnel costs for this absence were calculated to amount to a total of approximately 63,000 € (Table 1).

**Table 1:** Total estimated cost of leave among staff affected by COVID-19

Type of staff	Confirmed COVID-19 Phase I + II	Confirmed COVID-19 Phase I (Outbreak and tracing)	Confirmed COVID-19 Phase II (Prevent-Covid protocol-implemented)	Days absent	Personnel costs (223 working days in 2020)
Nurses	6/10 (60%)	5/10 (50%)	1/10 (10%)	110 (*n* = 6) Average: 18	27,500 €
Physicians	2/7 (28%)	2/7 (28%)	0/7 (0%)	35 (*n* = 2) Average: 18	19,100 €
Study staff	1/5 (20%)	1/5 (20%)	0/5 (0%)	29 (*n* = 1) Average: 29	7300 €
Administrative staff	1/5 (20%)	1/5 (20%)	0/5 (0%)	13 (*n* = 1) Average: 13	3000 €
Cleaning staff	1/1 (100%)	1/1 (100%)	0/1 (0%)	32 (*n* = 1) Average: 32	6200 €
	11/28 (39%)	10/28 (36%)	1/28 (4%)	219 days Average: 20	63,100 €

## Discussion

The SARS-CoV-2 pandemic is probably one of the biggest health and economic burdens of the century. As the coronavirus continues to mutate and spread globally, millions of humans are still affected [20]. More than 24 months after the beginning of the outbreak in China in 2019, and despite the rollout of vaccines, the world is still dealing with increased numbers of confirmed cases in different countries and continents spread throughout the world. This is the first pandemic that is recognized with such global impact in this century, yet scientists postulate that in the future more will come [21]. Therefore, it is important to clearly describe successful prevention measures implemented in various settings [22].

Even before such recommendations were implemented as standard of care, the data from Phase 1 of this study underscore the importance of proactive testing, contact tracing, wearing medical masks and adhering to social distancing. Combining these interventions, we were able to contain the outbreak and, to this day, have had no further outbreaks that have spread within our outpatient clinic. In fact, we believe that due to the implementation of risk reduction measures in Phase II, we were further able to identify additional cases in the early, and often asymptomatic, phase. In this manner, we could also prevent further spread of the disease to our patients and among our staff. As we performed regular screening by PCR-tests during phase II, before this became standard procedure across the clinic and at our own costs, we were able to quickly identify and isolate cases and continue to adhere to these measure as they are lifted within the community. This is particularly important as in later stages of the pandemic, it still remains difficult to identify mild or asymptomatic individuals and breakthrough infections and therefore this practice has proven effective to date [23, 24]. We believe that our decision to invest early in regular screening should be a standard procedure to curtail outbreaks of potential future contagious diseases, especially in the setting of oncology outpatient clinics. In fact, based on the successful containment of the outbreak in our unit, similar hygiene and social distancing standards as applied during Phase I of this study were adopted as standard practice recommendations throughout the University hospital, although these standards were not required by state mandate. At a later time point however, the German Society of Hematology added most of these measures to their recommendations.

We therefore recommend that these measures, including the proactive- asymptomatic testing as part of routine care, become standard procedures at oncology outpatient clinics. The addition of the symptom tracker per App supported us for symptom tracing and identification among the subset (*n* = 39) who agreed to download the app. However, due to the low percent of patients who participated (7% of the 525 patients) and the fact that the average acceptance rate among those who downloaded the app varied greatly, we do not deem that this measure is essential for the prevention of the spread of viral diseases at this point in time. The serological analyses, while scientifically interesting, did not provide any additional relevant data with respect to disease prevention measures in our study. Likewise, as the clinical utility of antibody testing remains unclear with respect to infection transmission and rates, it is not possible to make a recommendation as to the role of antibody testing within the context of viral disease prevention among oncology patients.

It is notable that, although we chose to invest in PCR testing, the investment potentially offset the cost of further absences caused by sick leave. Regardless of the potential socio-economic benefit, the investment in patient safety is well worth it.

The fact that we were able to perform virus genome sequencing that helped to trace transmission chains is one strength of this study. Furthermore, we were able to recruit a large majority of our patients at the peak of the first wave which helped to give a good indication if the measures were successful. We also consider the fact that we could collect data regarding the financial burden to be a strength. However, our study was limited by the small sample size and the fact that we relied on participation from both the HCPs and patients to report the PCR-test results to us if performed outside of the hospital. It could therefore be possible that we were not informed about further positive cases among the cohort during the duration of the study. Lastly, the fact that this was an observational study and we therefore have no control group as comparison could be considered a limitation.

In conclusion, limited research and reports regarding protocols for the care of outpatient cancer patients during an infectious pandemic exist. Our data underscores the importance of identification of outbreaks in the early phases and illustrates that the identification of potential carriers is essential to ensure the safety of this vulnerable population. Our experience also showed that a quick and methodical response could play a big role in interrupting the infection chain and providing a safe environment where the risk of infection remained as low as possible. As no further outbreaks have occurred since the implementation of the described measures, we believe this is mostly due to routine screening which enabled us to identify and isolate asymptomatic or pre-symptomatic cases. In preparation for future pandemics, outpatient cancer units should all have strict standards of practice in place. Additionally, all employees should be aware of these protocols and be prepared to implement them without hesitation. While more prospective controlled studies are needed to confirm these results, we also recommend that universal hygiene precautions should be combined with proactive routine testing and integrated into standard care. Although these measures may require investment of both personnel and financial resources, our data indicates that the potential benefits outweigh the costs.

## Data Availability

The datasets generated during and/or analyzed during the current study are available from the corresponding author on reasonable request.

## Abbreviations

HCPs: Health-care professionals
PHEIC: Public health emergency of international concern
RT-PCR: Reverse-transcription polymerase chain reaction assay test
RT-qPCR: Reverse-transcription quantitative polymerase chain reaction assay test
WHO: World Health Organization

## Code availability

CANKADO is approved as an active Class I medical device within the European Union (registration number DE/CA59/11976/2017) and is compliant with the FDA classification for Mobile Medical Devices (2015 Appendix B).
